# Trait emotional experience in individuals with schizophrenia and youth at clinical high risk for psychosis

**DOI:** 10.1192/bjo.2019.64

**Published:** 2019-09-10

**Authors:** Claire I. Yee, Gregory P. Strauss, Daniel N. Allen, Claudia M. Haase, David Kimhy, Vijay A. Mittal

**Affiliations:** Postdoctoral Fellow, Department of Psychology and School of Education and Social Policy, Northwestern University, USA; Assistant Professor, Department of Psychology, University of Georgia, USA; Director of Clinical Training, Department of Psychology, University of Nevada, USA; Assistant Professor, School of Education and Social Policy, Northwestern University, USA; Associate Professor, Department of Psychiatry, Icahn School of Medicine at Mount Sinai, USA; Associate Professor, Department of Psychology, Northwestern University, USA

**Keywords:** Psychosis, prodrome, emotion, negative symptoms

## Abstract

**Background:**

Disturbances in trait emotions are a predominant feature in schizophrenia. However, less is known about (a) differences in trait emotion across phases of the illness such as the clinical high-risk (CHR) phase and (b) whether abnormalities in trait emotion that are associated with negative symptoms are driven by primary (i.e. idiopathic) or secondary (e.g. depression, anxiety) factors.

**Aims:**

To examine profiles of trait affective disturbance and their clinical correlates in individuals with schizophrenia and individuals at CHR for psychosis.

**Method:**

In two studies (sample 1: 56 out-patients diagnosed with schizophrenia and 34 demographically matched individuals without schizophrenia (controls); sample 2: 50 individuals at CHR and 56 individuals not at CHR (controls)), participants completed self-report trait positive affect and negative affect questionnaires, clinical symptom interviews (positive, negative, disorganised, depression, anxiety) and community-based functional outcome measures.

**Results:**

Both clinical groups reported lower levels of positive affect (specific to joy among individuals with schizophrenia) and higher levels of negative affect compared with controls. For individuals with schizophrenia, links were found between positive affect and negative symptoms (which remained after controlling for secondary factors) and between negative affect and positive symptoms. For individuals at CHR, links were found between both affect dimensions and both types of symptom (which were largely accounted for by secondary factors).

**Conclusions:**

Both clinical groups showed some evidence of reduced trait positive affect and elevated trait negative affect, suggesting that increasing trait positive affect and reducing trait negative affect is an important treatment goal across both populations. Clinical correlates of these emotional abnormalities were more integrally linked to clinical symptoms in individuals with schizophrenia and more closely linked to secondary influences such as depression and anxiety in individuals at CHR.

**Declaration of interest:**

None.

Emotional abnormalities have been a core aspect of schizophrenia since the earliest conceptualisations of the disorder. For example, Kraepelin described individuals with schizophrenia as having ‘a weakening of those emotional activities which permanently form the mainsprings of volition’.^1^ Bleuler similarly emphasised that individuals with schizophrenia were ‘disturbed in a number of ways, but above all the breakdown of the emotion’.^1^ Modern empirical studies support these early observations by documenting abnormalities in a variety of emotional processes, such as facial and vocal expressivity, emotion perception, emotion regulation and trait emotional experience;^2–6^ however, some emotional processes appear to be intact (e.g. state positive emotional experience).^7–9^ Importantly, when abnormalities are present in the aforementioned processes, they are consistently linked to poor clinical outcomes, such as increased severity of positive, negative and disorganised symptoms, poor community-based functional outcome and lower quality of life.^10–14^

Although emotional abnormalities are clinically important in the active phases of schizophrenia, it is unclear whether they are also associated with liability for the illness. Psychotic disorders are typically preceded by a prodromal (i.e. pre-illness) phase characterised by functional decline and subtle attenuated hallucinations and delusions that progressively worsen over the course of several months to years.^15,16^ This period is of interest both as a window for investigating processes involved in illness onset and as a potential point of intervention and prevention.^17,18^ Documenting the nature of emotional abnormalities among individuals at clinical high risk (CHR) for psychosis is critical for enhancing early identification and intervention efforts.^19,20^

## Trait emotional experience studies in schizophrenia

Trait emotional experience (i.e. dispositional tendencies to experience positive or negative emotions in response to life's events in enduring and predictable ways^21^) is a critical aspect of emotionality that has received relatively little attention in the CHR literature. Studies administering trait emotional experience questionnaires to healthy individuals typically identify two distinct dimensions that are only moderately correlated: positive and negative affect.^22^ These dimensions predict mental and physical health for a number of conditions.^23,24^ A large number of studies administering trait questionnaires to individuals with schizophrenia indicate that they report lower levels of general positive affect and higher levels of general negative affect than individuals without schizophrenia (for a review see Horan *et al*^11^). Differences in discrete trait emotions can also be observed between individuals with schizophrenia and those without. For example, individuals with schizophrenia report lower levels of trait joy and higher levels of trait fear and disgust and these differences are greatest for those with high levels of anhedonia and blunted affect.^25^ In a more general vein, low trait positive affect in schizophrenia has been linked to lower quality of life, smaller social networks, depression, anxiety and anhedonia.^26–28^ The association between lower levels of positive affect and negative symptoms is particularly strong across studies.^29–32^ Conversely, high trait negative affect in schizophrenia has been linked to worse coping, occupational and social functioning, lower satisfaction with life, as well as increased smoking, alcohol and drug use.^28,33–36^

To our knowledge, only one study has evaluated trait positive and negative affect in individuals at CHR for psychosis. Seo and colleagues^37^ found that individuals at CHR reported lower levels of trait positive and higher levels of trait negative affect. Moreover, only negative symptoms predicted individual differences in both higher negative and lower positive trait affect.

Taken together, these findings suggest that trait affect may be critically related to negative symptoms in both individuals with schizophrenia and individuals at CHR for psychosis. However, negative symptoms are heterogeneous and it is possible for two participants to receive exactly the same score on a rating scale for different reasons. In schizophrenia, it is well-documented that negative symptoms can reflect both primary (i.e. idiopathic) and secondary (e.g. depression, anxiety) factors.^38^ Individuals with schizophrenia with primary and enduring negative symptoms have clinical, biological and emotional profiles that are distinct from individuals with secondary negative symptoms or those with low levels of negative symptoms.^39–42^ Secondary negative symptom sources (e.g. depression, anxiety) are also prominent in individuals at CHR^43^ and are strongly associated with negative symptoms;^44^ however, secondary negative symptoms tend to be transient, resolving with alleviation of the secondary influence. In contrast, primary negative symptoms tend to be persistent during the prodromal period, and can be identified at rates comparable to those in chronic schizophrenia (~33%).^45^ These findings suggest that clarifying the extent to which trait affective abnormalities are linked to primary and secondary negative symptoms across phases of illness is of critical importance because it would suggest how treatment efforts would be best directed at each stage.

## The current study

The current study administered a trait emotional experience questionnaire and measures of clinical outcomes to cross-sectional samples of out-patients with schizophrenia and at CHR for psychosis to examine profiles of trait affective disturbance and their clinical correlates. The following hypotheses were tested: (a) both individuals with schizophrenia and those at CHR for psychosis would evidence diminished trait positive affect and elevated trait negative affect compared with control groups; (b) lower trait positive affect would be associated with increased severity of negative symptoms and poorer community-based functional outcome across both the sample with schizophrenia and the sample at CHR; and (c) higher trait negative affect would be associated with increased severity of positive and negative symptoms and poorer community-based functioning across both the samples with schizophrenia and the sample at CHR.

Prior psychosis studies have administered measures that were not amenable to examining the role of discrete emotions; however, examination of discrete emotions is an important extension because individual positive (e.g. joy, interest) and negative (e.g. sadness, fear) emotions can have unique effects on physiology, health and behaviour.^46–48^ We therefore conducted exploratory analyses to determine whether individuals with schizophrenia and those at CHR showed different patterns of abnormalities on discrete trait emotions. Exploratory analyses were also conducted to determine whether associations between negative symptoms and trait affect reflected secondary factors in the samples with schizophrenia and at CHR. These analyses focused on depression, anxiety and antipsychotics as the most common secondary factors that are prevalent in both populations with schizophrenia and those at CHR.^38,43^ Negative symptoms do not reflect a singular construct,^49^ but rather two broad dimensions reflecting diminished expressivity and diminished motivation and pleasure, or five discrete domains reflecting anhedonia, avolition, asociality, alogia and blunted affect.^50–52^ Therefore, exploratory analyses examined associations between trait affect and the two broad negative symptom dimensions, as well as the five domains.

## Method

The samples used for the study originate from two separate studies. Both studies were approved by the local institutional review boards, and written informed consent or assent was obtained from all participants.

### Sample 1

#### Participants

Sample 1 comprised 56 individuals meeting DSM-IV-TR^53^ criteria for schizophrenia (79%) or schizoaffective disorder (21%) and 34 individuals without schizophrenia (controls). Individuals with schizophrenia were recruited from out-patient psychiatric mental health facilities and evaluated during periods of clinical stability, as determined by a minimum of 4 weeks of consistent medication dose and type or, in the absence of medication, deemed clinically stable by their treating clinician, as indicated by no significant changes in symptoms or functioning within the previous month. Consensus diagnosis was established via a best-estimate approach based on psychiatric history and multiple interview and subsequently confirmed using the Structured Clinical Interview for DSM-IV (SCID-I^54^). All individuals with schizophrenia met DSM-IV current diagnostic criteria for schizophrenia or schizoaffective disorder; 93% were prescribed antipsychotic medications at the time of testing. Individuals without schizophrenia were recruited through posted advertisements and word of mouth among enrolled participants. All individuals without schizophrenia underwent a screening interview, including the SCID-I, and did not meet lifetime criteria for a psychotic disorder or any current Axis I disorder. Individuals without schizophrenia also had no self-reported family history of psychosis. The SCID-I was used to determine that no participants met DSM-IV criteria for substance misuse or dependence over the previous 6 months. All participants were also screened for lifetime neurological disorders and were free from neurological conditions (e.g. traumatic brain injury, epilepsy). Demographic details for sample 1 are given in Table 1.

**Table 1:** Demographic details for sample 1

	Individuals with SZ	Individuals without SZ	Total
Age, mean (s.d.)	39.30 (9.72)	37.79 (12.43)	38.73 (10.78)
Gender, *n*			
Male	30	13	43
Female	26	21	47
Total	56	34	90
Education, years: mean (s.d.)	11.88 (1.99)	13.60 (1.42)	12.53 (1.98)
Antipsychotics, in CPZeq/day: mean (s.d.)	773.76 (637.10)		
DES positive affect, mean (s.d.)	1.05 (0.25)	1.05 (0.15)	1.05 (0.22)
DES negative affect, mean (s.d.)	0.85 (0.27)	0.70 (0.19)	0.80 (0.26)
BPRS anxiety, mean (s.d.)	3.18 (1.58)	–	–
BPRS depression, mean (s.d.)	1.89 (1.45)	–	–
GAF, mean (s.d.)	31.94 (11.62)	–	–
SAPS, mean (s.d.)^a^	33.72 (18.68)	–	–
SANS, mean (s.d.)^a^	43.71 (28.45)	–	–
Diminished expression, mean (s.d.)	1.22 (1.06)	–	–
Diminished motivation/pleasure, mean (s.d.)	1.50 (1.17)	–	–
Blunted affect, mean (s.d.)	1.32 (1.24)	–	–
Alogia, mean (s.d.)	1.11 (1.09)	–	–
Avolition, mean (s.d.)	1.4 (1.11)	–	–
Anhedonia, mean (s.d.)	1.34 (1.37)	–	–
Asociality, mean (s.d.)	1.75 (1.43)	–	–

SZ, schizophrenia; DES, Differential Emotions Scale IV-A; CPZeq, chlorpromazine equivalents; BPRS, Brief Psychiatric Rating Scale; GAF, Global Assessment of Functioning; SAPS, Scale for the Assessment of Positive Symptoms; SANS, Scale for the Assessment of Negative Symptoms.

a. Symptom domains are represented as the sum score of items for each specific category (positive and negative).

#### Measures

##### Clinical interviews

The Scale for the Assessment of Positive Symptoms (SAPS)^55^ and Negative Symptoms (SANS)^56^ were administered by clinical psychology doctoral students trained to reliability standards (α > 0.80) to measure participants' positive and negative symptoms. The Brief Psychiatric Rating Scale (BPRS)^57^ was used to assess anxiety and depression. The Global Assessment of Functioning (GAF) score was used as a gross measure of global functioning.

##### Trait emotional experience

The Differential Emotions Scale IV-A (DES)^58^ is a 30-item self-report measure designed to assess the frequency with which individuals experience discrete basic emotions in everyday life. The questionnaire yields ten basic emotion scales (each measured by three items), with three scales representing positive emotions (joy, interest and surprise), which are averaged to measure positive affect (α = 0.73), and seven representing negative emotions (sadness, anger, fear, disgust, contempt, guilt and shame), which are averaged to measure negative affect (α = 0.91). Participants are required to identify the frequency with which they experience 30 emotions on a 5-point scale (1, rarely or never experienced; 5, experienced very often). DES items reflect verbal labels commonly applied to identify facial expressions, as well as current theories of basic emotion, which describe emotions in terms of discrete categories.^59^ This measure has been used in previous schizophrenia investigations, and demonstrated differences in experience among discrete emotions in individuals with and without schizophrenia.^25^

### Sample 2

#### Participants

Sample 2 comprised a group of 50 adolescents and young adults aged between 13 and 21 years (mean 19.06, s.d. = 2.28) at CHR for psychosis and 56 matched individuals not at CHR (controls) recruited to the Adolescent Development and Preventive Treatment (ADAPT) research programme using internet, newspaper and public transportation advertisements, email postings and community professional referrals. Individuals at CHR in the present study met Structured Interview for Prodromal Syndromes (SIPS) criteria for a prodromal syndrome, defined by moderate-to-severe but not psychotic levels of positive symptoms (rated between 3 and 5 on a 6-point scale) or a decline in global functioning accompanying the presence of schizotypal personality disorder or a family history of schizophrenia.^60^ Family history of psychosis was obtained by asking participants whether any first-degree family members had been diagnosed with a psychotic disorder. In most cases, family history was corroborated by another family member of the participant. Exclusion criteria consisted of head injury, the presence of a neurological disorder and lifetime substance dependence. The presence or lifetime history of an Axis I psychotic disorder at baseline was also an exclusion criterion for control and CHR status. See Table 2 for demographic information.

**Table 2:** Demographic details for sample 2

	Individuals at CHR	Individuals not at CHR	Total
Age, mean (s.d.)	18.94 (1.82)	19.16 (2.62)	19.06 (2.28)
Gender, *n*			
Male	29	22	51
Female	21	34	55
Total	50	56	106
Parental education, years: mean (s.d.)^a^	15.67 (2.30)	15.73 (2.63)	15.6 (2.49)
Participants taking antipsychotics, *n*	9	0	9
Positive affect, mean (s.d.)	2.37 (0.73)	2.88 (0.57)	2.64 (0.68)
Negative affect, mean (s.d.)	1.79 (0.85)	0.81 (0.76)	1.27 (0.93)
BAI, mean (s.d.)	20.32 (12.18)	5.65 (6.65)	12.51 (12.08)
BDI, mean (s.d.)	1.29 (11.41)	3.21 (4.40)	12.51 (12.08)
GFS: Social, mean (s.d.)	6.88 (1.65)	8.73 (0.68)	7.86 (1.53)
GFS: Role, mean (s.d.)	6.96 (1.49)	8.65 (0.70)	7.88 (1.41)
SIPS positive, mean (s.d.)^b^	11.78 (4.62)	0.34 (0.84)	5.62 (6.53)
SIPS negative, mean (s.d.)^b^	9.58 (7.37)	0.23 (0.54)	4.52 (6.83)
Diminished emotion, mean (s.d.)	1.78 (1.51)	0.05 (0.14)	0.84 (1.34)
Diminished volition, mean (s.d.)	1.64 (1.37)	0.03 (0.12)	0.77 (1.23)

CHR, clinical high risk for psychosis; BAI, Beck Anxiety Inventory; BDI, Beck Depression Inventory; GFS, Global Functioning Scale; SIPS, Structured Interview for Prodromal Syndromes.

a. Parental education is the average of the mother's and father's education.

b. Symptom domains are represented as the total score of items for each specific category.

#### Measures

##### Clinical interviews

Interviews were administered by expert raters trained to reliability standards (α > 0.80). The SIPS was administered at baseline to diagnose a prodromal syndrome and assess symptoms.^60,61^ A total sum score for the positive, negative and disorganised symptom domains is used as an indicator of the respective dimensions of symptomatology.

Depression and anxiety were assessed using the Beck Depression Inventory (BDI)^62^ and the Beck Anxiety Inventory (BAI).^63^

The Global Functioning Scale: Social (GFS-S) and Role (GFS-R)^64^ were used to assess community-based functional outcome (e.g. number of friends, how often the individual engages in social activity, performance at school, work). A score is given on a 1–10 scale, with 1 indicating low levels of functioning and 10 indicating very high levels of functioning.

##### Trait emotional experience

The Modified Differential Emotions Scale (mDES)^65^ was used to measure trait emotional experience. Participants rated the extent to which they felt each of 20 emotions on average or in general on a 5-point scale from 0 (not at all) to 4 (extremely). The mDES differs from the DES in that it measures each emotion with a single item, thus increasing the number of discrete positive emotions from three to ten. The ten positive emotions (amusement, awe, content, joy, gratitude, hope, interest, love, pride and surprise) were averaged together to form the positive affect composite (α = 0.88). The negative affect composite averaged scores for anger, shame, fear, disgust, embarrassment, guilt, sadness and contempt (α = 0.93).

### Data analytic strategy

Repeated measures ANOVAs were used to test differences in the pattern of self-reported emotional experience among clinical and control samples. Each ANOVA had two groups (schizophrenia/CHR, control) with two emotion dimensions (positive, negative). Significant interactions were followed up with one-way ANOVAs and within-group paired samples *t*-tests.

Correlations were analysed to examine the relationship between emotionality, symptoms and functioning in the individuals with schizophrenia and those at CHR. In the first set of correlations, emotionality included the positive and negative affect composites, symptoms included positive and negative totals (and disorganised for sample 2) and functionality included the GAF for sample 1 and GFS-S and GFS-R for sample 2. In the second set of correlations, negative symptoms were broken into two subsets: two subdimensions (i.e. diminished expression and diminished motivation in individuals with schizophrenia, and diminished emotion and diminished volition in individuals at CHR),^66,67^ and the five National Institute of Mental Health (NIMH) negative symptom domains (i.e. anhedonia, avolition, asociality, alogia and blunted affect).^68^ To test the extent to which relationships in this second set of correlations were influenced by secondary factors, partial correlations were run controlling for depression and anxiety (and antipsychotics in sample 1). In sample 2, all partial correlations were re-run excluding individuals on antipsychotics. No correlations changed significantly in magnitude or direction when excluding these individuals. The Benjamini–Hochberg correction procedure was used for each set of correlations to control for multiple correlation tests.^69^ Results of the procedure are stated at the end of each correlation section.

## Results

### Group differences in trait emotional experience

#### Affect differences between individuals with and without schizophrenia

**Fig. 1:**
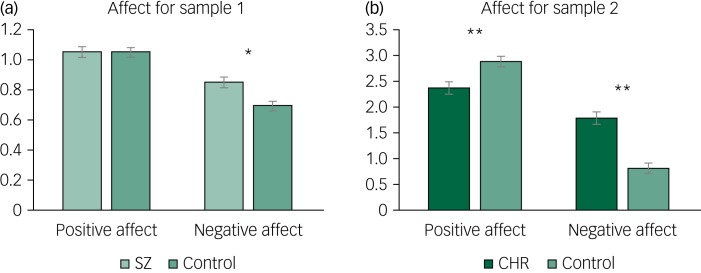
Differences in positive and negative affect in samples 1 and 2.

A repeated-measures ANOVA was conducted to test for group differences in trait emotional experience between individuals with and without schizophrenia. There was a statistically significant group × emotion interaction, in addition to a main effect of group and a within-participants effect of emotion. Follow-up one-way ANOVAs showed higher levels of negative emotion in individuals with schizophrenia compared with individuals without schizophrenia, but no group differences in positive emotion. Paired-samples *t*-tests showed higher levels of positive than negative emotion within each group (Table 3; Fig. 1).

Bars in each graph represent standard error. SZ, individuals with schizophrenia; CHR, individuals at clinical high risk for psychosis.

**Table 3:** Omnibus ANOVA and *post hoc* results for group affect differences

	Test statistic	*P*	Cohen's *d*
Sample 1: positive *v.* negative affect			
Group	*F*_1,80_ = 4.06	0.05	0.46
Emotion	*F*_1,80_ = 55.28	<0.001	1.67
Group × emotion	*F*_1,80_ = 4.06	0.04	0.46
*Post hoc* one-way ANOVAs			
Positive	*F*_1,80_ < 0.001	0.99	<0.01
Negative	*F*_1,80_ = 7.42	<0.01	0.64
*Post hoc* paired-samples *t*-tests			
SZ: positive *v.* negative	*t*_53_ = 4.27	<0.001	0.59
Control: positive *v.* negative	*t*_29_ = 6.66	<0.001	1.24
Sample 2: positive *v.* negative affect			
Group	*F*_1,103_ = 7.10	<0.01	0.55
Emotion	*F*_1,103_ = 137.05	<0.001	2.30
Group × emotion	*F*_1,103_ = 43.00	<0.001	1.31
*Post hoc* one-way ANOVAs			
Positive	*F*_1,103_ = 16.38	<0.001	0.80
Negative	*F*_1,103_ = 38.31	<0.001	1.22
*Post hoc* paired-sample *t*-tests			
CHR: positive *v.* negative	*t*_50_ = 3.04	<0.01	0.43
Control: positive *v.* negative	*t*_55_ = 16.26	<0.001	2.19

SZ, individuals with schizophrenia; CHR, individuals at clinical high risk for psychosis.

#### Affect differences between individuals at CHR and not at CHR

A repeated-measures ANOVA showed a significant group × emotion interaction, in addition to a main effect of group and a within-participants effect of emotion. Follow-up one-way ANOVAs showed lower levels of positive and higher levels of negative affect in individuals at CHR for psychosis compared with individuals not at CHR. Paired-samples *t*-tests showed higher levels of positive than negative emotion within each group (Table 3; Fig. 1).

#### Exploratory discrete emotion differences between both samples

**Fig. 2:**
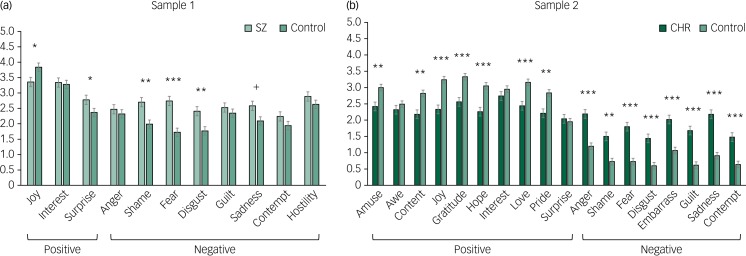
Differences in discrete emotions in samples 1 and 2.

Across samples, both repeated-measures ANOVAs showed a significant group × emotion interaction and a within-participants effect of emotion. Follow-up one-way ANOVAs showed that in sample 1, two of the three positive emotions (i.e. joy and surprise) showed significant differences between individuals with and without schizophrenia. For joy, individuals with schizophrenia reported lower joy than individuals without schizophrenia, whereas individuals with schizophrenia reported more surprise compared with individuals without schizophrenia. However, after correcting for multiple tests, these two differences between groups were not significant. Four of the eight negative emotions (i.e. shame, fear, disgust and sadness) showed marginal or significant differences (Table 4), whereas the other four (i.e. anger, guilt, contempt and hostility) showed no significant differences between individuals with and without schizophrenia. In sample 2, seven of the ten positive emotions (i.e. amusement, contentment, joy, gratitude, hope, love and pride) and each of the eight negative emotions (i.e. anger, shame, fear, disgust, embarrassment, guilt, sadness and contempt) showed significant differences between individuals at and not at CHR for psychosis (Fig. 2). In all cases, individuals at CHR reported more negative emotions than individuals not at CHR. All group differences remained unchanged after correcting for multiple testing, except for the findings for joy and surprise in sample 1.

In sample 1, group differences for joy and surprise did not meet multiple testing correction criteria. SZ, individuals with schizophrenia; CHR, individuals at clinical high risk for psychosis.

**Table 4:** Omnibus ANOVAs and *post hoc* results for discrete trait emotions

	Test statistic	*P*	Cohen's *d*
Sample 1			
Group	*F*_1,80_ = 6.61	0.01	0.60
Emotion	*F*_10,800_ = 30.38	<0.001	1.29
Group × emotion	*F*_10,800_ = 4.94	<0.01	0.52
*Post hoc* one-way ANOVAs			
Joy	*F*_1,80_ = 4.47	0.04^a^	0.49
Interest	*F*_1,80_ = 0.12	0.73	0.08
Surprise	*F*_1,80_ = 6.18	0.02^a^	0.58
Anger	*F*_1,80_ = 0.41	0.52	0.15
Shame	*F*_1,80_ = 11.60	<0.01	0.80
Fear	*F*_1,80_ = 18.18	<0.001	1.00
Disgust	*F*_1,80_ = 9.15	<0.01	0.71
Guilt	*F*_1,80_ = 0.66	0.42	0.19
Sadness	*F*_1,80_ = 3.81	0.06	0.46
Contempt	*F*_1,80_ = 1.82	0.18	0.32
Hostility	*F*_1,80_ = 2.44	0.12	0.37
Sample 2			
Group	*F*_1,103_ = 2.64	0.11	0.32
Emotion	*F*_17,1751_ = 67.15	<0.001	1.62
Group × emotion	*F*_1,1751_ = 21.25	<0.001	0.91
*Post hoc* one-way ANOVAs			
Amusement	*F*_1,103_ = 12.23	<0.01	0.69
Awe	*F*_1,103_ = 0.68	=0.41	0.16
Contentment	*F*_1,103_ = 2.64	<0.01	0.32
Joy	*F*_1,103_ = 30.47	<0.001	1.09
Gratitude	*F*_1,103_ = 19.75	<0.001	0.88
Hope	*F*_1,103_ = 20.65	<0.001	0.90
Interest	*F*_1,103_ = 1.71	=0.19	0.26
Love	*F*_1,103_ = 15.72	<0.001	0.78
Pride	*F*_1,103_ = 9.84	<0.01	0.62
Surprise	*F*_1,103_ = .21	=0.65	0.09
Anger	*F*_1,103_ = 24.89	<0.001	0.98
Shame	*F*_1,103_ = 15.58	<0.001	0.78
Fear	*F*_1,103_ = 29.04	<0.001	1.06
Disgust	*F*_1,103_ = 17.38	<0.001	0.82
Embarrassment	*F*_1,103_ = 19.11	<0.001	0.86
Guilt	*F*_1,103_ = 28.20	<0.001	1.05
Sadness	*F*_1,103_ = 39.64	<0.001	1.24
Contempt	*F*_1,103_ = 17.58	<0.001	0.83

a. The finding did not meet multiple testing correction criteria.

### Trait emotional experience and clinical and functional outcomes

#### Affect relationships to symptoms and functioning in individuals with schizophrenia

**Fig. 3:**
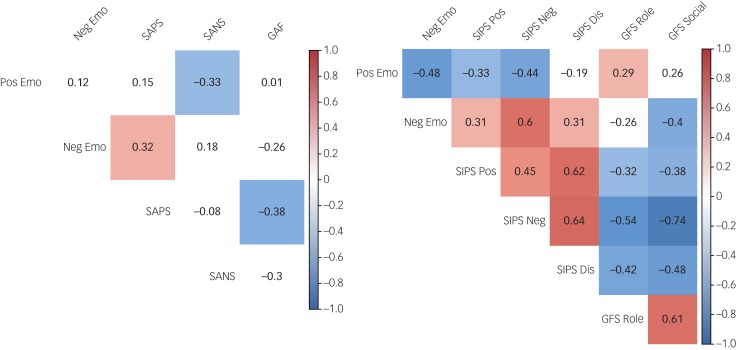
Correlations between affect, clinical symptoms and functioning in samples 1 and 2.

Pearson correlations were conducted to investigate the degree to which positive and negative emotions were related to clinical symptoms and functional outcomes in the individuals with schizophrenia. Lower levels of positive affect were related to higher levels of negative symptoms but were not significantly related to positive symptoms. Higher levels of negative affect were related to higher levels of positive symptoms and were unrelated to negative symptoms. Neither type of emotion was directly related to functioning (Fig. 3). All relationships remained unchanged after correcting for multiple correlation tests.

Pos, positive; Neg, negative; Emo, affect; SAPS, Scale for the Assessment of Positive Symptoms; SANS, Scale for the Assessment of Negative Symptoms; GAF, Global Assessment of Functioning; SIPS, Structured Interview for Prodromal Syndromes; Dis, disorganized symptoms; GFS, Global Functioning Scale.

#### Affect relationships to symptoms and functioning in individuals at CHR

Correlations in the individuals at CHR for psychosis showed that lower levels of positive affect were related to higher levels of both positive and negative symptoms. Higher levels of negative affect were related to higher levels of positive, negative and disorganised symptoms. Positive affect was positively associated with social functioning, and negative affect was negatively associated with negative role functioning. Higher levels of SIPS symptoms were all related to lower levels of social and role functioning (Fig. 3, right panel). All relationships remained unchanged after correcting for multiple correlation tests.

#### Exploratory affect relationships to negative symptom subtypes in individuals with schizophrenia and at CHR

To further explore the relationship between affect and negative symptoms in individuals with schizophrenia, negative symptoms were decomposed in two sets of analyses: the two subdimensions of expressivity and motivation, and the five NIMH domains. When examining the two subdimensions of negative symptoms, strong relationships between diminished motivation and both positive affect and negative affect emerged, whereas expressivity was only marginally related to positive affect and unrelated to negative affect. When examining negative symptoms using the NIMH domains, correlation analyses showed that positive affect was significantly negatively related to all NIMH domains except alogia, and only marginally related to blunted affect. The relationship between negative affect and the five domains seemed more complex, with negative affect being significantly related only to anhedonia and marginally related to alogia and asociality (supplementary Table 1 available at https://doi.org/10.1192/bjo.2019.64). All relationships remained unchanged after correcting for multiple correlation tests.

The same two-dimension approach for negative symptoms in individuals at CHR for psychosis showed that higher levels of both dimensions of negative symptoms (i.e. diminished expressivity and diminished motivation) were related to lower levels of positive affect and higher levels of negative affect. In analysis of the individual SIPS items, every item was related to higher levels of negative affect, whereas positive affect was related only to the avolition, emotion expression and emotion experience items (supplementary Table 2). All relationships remained unchanged after correcting for multiple correlation tests.

### Secondary negative symptom influences on trait emotional experience

#### Relationships between affect and negative symptoms controlling for anxiety, depression and antipsychotics in individuals with schizophrenia

After controlling for anxiety, depression and antipsychotic medication, only the relationship between positive affect and negative symptoms remained significant in individuals with schizophrenia. For the five NIMH domains, only the relationship between positive affect and the NIMH domains of avolition, anhedonia and asociality remained significant (supplementary Table 3). All relationships remained unchanged after correcting for multiple correlation tests.

#### Relationships between affect and negative symptoms controlling for anxiety and depression in individuals at CHR

After controlling for anxiety and depression, only the relationship between negative affect and diminished motivation remained significant in individuals at CHR for psychosis. Similarly, only the relationships between negative affect and the individual SIPS components remained significant when controlling for anxiety and depression (supplementary Table 4). All relationships remained unchanged after correcting for multiple correlation tests.

## Discussion

In this study we observed that clinical samples of individuals with schizophrenia and individuals at CHR for psychosis showed differences from their respective controls in trait emotional experiences. Furthermore, differences in trait emotional experiences were closely linked to clinical symptoms and functional outcomes. This study was the first of its kind to examine differences in trait emotion across a wide set of emotions in individuals with schizophrenia and individuals at CHR. In individuals with schizophrenia, the relationship between the positive-emotion deficit and negative symptoms remained after controlling for secondary influences of depression and anxiety. In individuals at CHR, it is noteworthy that controlling for depression and anxiety eliminated the relationship between the positive-emotion deficit and negative symptoms. The following paragraphs discuss the implications of these findings.

### Levels of trait positive and negative affect in schizophrenia

Individuals with schizophrenia reported higher levels of trait negative affect compared with individuals without schizophrenia, converging with prior literature,^11,25^ including findings by Cohen & Minor,^7^ who showed that individuals with schizophrenia are likely to report aversive reactions to positive and negative stimuli. The present study showed that negative affect was driven by disgust, fear and shame in particular, converging with findings by Birchwood & colleagues,^70^ who showed that fear and shame specifically may drive avoidance behaviour in psychosis (and disgust has similar avoidance functions).^71^ No differences were found for other negative emotions (i.e. anger, guilt, contempt and hostility) or for global positive affect in the groups with and without schizophrenia. However, examining specific positive emotions revealed preliminary opposing effects for joy (which was reduced) and surprise (which was elevated) in individuals with schizophrenia. Although these results did not stand up to multiple test correction, they directionally support existing evidence of reduced positive trait affect in individuals with schizophrenia.^11,25,72^ When interpreting the directionally elevated levels of surprise in individuals with schizophrenia, it is important to consider that individuals typically do not perceive surprise as emotionally positive but rather as ambiguous or even negative.^73^

### Generalised emotion deficits in individuals at CHR

Individuals at CHR for psychosis showed a broad array of abnormalities in trait positive and negative emotional experiences. Specifically, individuals at CHR reported lower levels of seven out of ten positive emotions (i.e. lower levels of amusement, contentment, joy, gratitude, hope, love and pride; no differences in awe, interest and surprise) and higher levels of all negative emotions compared with individuals not at CHR. These findings highlight that the emotion deficits in individuals at CHR are fairly generalised. It should be noted that exploratory analyses revealed that, for those at CHR who converted to a psychosis syndrome (*n* = 3, from the original group of 50) during the 2-year follow-up period, emotional alterations were in the same direction as for the rest of the original group at CHR, with directionally lower levels of positive emotions (mean 1.50, s.d. = 0.36) and higher levels of negative emotions (mean 1.92, s.d. = 0.29) compared with controls. Although the small number of individuals who developed a psychosis syndrome does not allow for direct comparisons with the general group at CHR, this is an important question for future studies with larger samples.

### Discrete emotions versus affective valence

Individuals with schizophrenia reported directionally different results for two of the three positive emotions (joy and surprise) and reported only some negative emotions as elevated. Although the differences in joy and surprise should be interpreted with caution, key significant findings remain (e.g. reports of higher levels of fear, shame and disgust, but not anger, guilt, contempt and hostility by individuals with schizophrenia compared with individuals without schizophrenia). These findings cumulatively emphasise the importance of moving beyond positive and negative affective valence toward considering discrete emotions (e.g. joy, surprise, fear, contempt), which may show unique patterns in individuals with schizophrenia and those at CHR for psychosis.

### Association of trait emotional experiences with symptoms and functioning

Individuals with schizophrenia and individuals at CHR showed differences in relationships between trait emotional experiences on the one hand and clinical symptoms and functioning on the other. For individuals with schizophrenia, only positive affect was associated with negative symptoms, and only negative affect was associated with positive symptoms. These results support the stronger relationships reported in the literature between the positive-emotion deficit and negative symptoms.^31,32,74^ Reviews of negative emotions and negative symptoms yield unreliable associations between the two.^11^ Exploring specific aspects of negative symptoms suggested that the absence of a relationship between negative affect and negative symptoms may be partly explained by the fact that only diminished motivation was related to abnormalities in both positive and negative affect. Additionally, analysing negative symptoms at the level of the five domains of negative symptoms (anhedonia, avolition, asociality, alogia and blunted affect) showed that positive affect was most closely associated with all five, again supporting the notion that negative symptoms are those most closely tied to the positive affect deficit. Although the absence of some of these relationships may be due to the small sample, these findings support existing literature that positive emotions are most closely associated with negative symptoms. Unlike prior research, neither type of affect predicted decreased functioning.^26,27^ However, both studies used measures that differed from previous research, as we used self-report rather than interviewer ratings and assessed the presence/absence of specific skills rather making normative assessments of functioning.

In individuals at CHR for psychosis, trait emotional experiences were highly interconnected with symptoms and functioning. Both positive and negative affect were linked to both positive and negative symptoms. Moreover, both positive and negative affect were linked to both negative symptom dimensions (i.e. diminished expressivity; diminished motivation and pleasure). When breaking these symptoms down into the five subdomains, negative affect was interconnected with more subdomains than positive affect. These findings add important specificity for studies showing links between emotions and negative symptoms.^5,37^ The breadth of interconnectedness between trait emotion, symptoms and functioning highlights not only the generalised pathology of emotion abnormalities in the population at CHR for psychosis, but also the importance of making emotions the target of interventions for improving functional outcomes.

### Influence of secondary factors on trait emotional experience

Secondary negative influences have not been studied in individuals with schizophrenia. This study found that controlling for secondary influences left only the relationship between positive affect and negative symptoms intact. The resilience of this relationship to these influences supports hypotheses that trait emotion differences, in particular positive-emotion deficits, may be more closely linked to the core of schizophrenia.^9,75^ Controlling for secondary influences in individuals at CHR for psychosis showed a strong relationship between negative affect and negative symptoms. These differences suggest that the emotion abnormalities (e.g. the positive-emotion deficit) seen in individuals at CHR may be driven more by a generalised psychopathology than the emotion abnormalities seen in individuals with schizophrenia. The concept of generalised psychopathology in individuals at CHR fits with recent findings that a high proportion of adolescents with anxiety and/or depression also display psychotic symptoms.^76^ It is possible that this high comorbidity rate in individuals at CHR reflects a close link between emotion dysregulation and psychosis early on. These findings also fit with other studies showing that individuals at CHR often report first with anxiety disorders or depression.^17,77^ The current findings suggest that a subset of individuals at CHR may have a high rate of generalised vulnerability for developing psychopathology. Identifying unique markers of these subgroups within the population of individuals at CHR can help isolate outcome groups earlier when young people first seek treatment.

### Limitations

The present findings should be viewed in light of several limitations of the study. The group of individuals with schizophrenia was relatively small and it may thus have been underpowered to detect smaller effects. Nevertheless, the group with schizophrenia in the current study was larger than every group with schizophrenia in one review of studies on emotion differences in schizophrenia^4^ and larger than over two-thirds of groups in another review of trait emotion in schizophrenia.^11^ Additionally, direct comparisons were limited between the individuals with schizophrenia and those at CHR for psychosis, as two different versions of the scale for measuring trait emotions were used for the two groups. Although exact parallels between the two groups cannot be drawn, both scales call attention to the importance of examining trait emotions at the discrete level, rather than always assuming generalisable effects for positive and negative affect. Literature showing that individuals with schizophrenia have a high degree of difficulty with emotional awareness suggests that caution should be used in interpreting strong differences between discrete emotions within the group with schizophrenia.^17,78^ However, the differences in some discrete emotions and not others found in the present study suggest that individuals with schizophrenia maintain some degree of differentiation. Future studies would benefit from examining how specific this level of differentiation is. Finally, differences in ages (e.g. adults with schizophrenia and adolescents and young adults at CHR) also limited direct comparisons between the two groups. However, the differences in age boost the external validity and generalisability of both samples.

### Future research

Taken together, these findings suggest that trait emotion abnormalities are an important consideration in schizophrenia. Although the stability of trait emotional experience abnormalities has yet to be determined, such research is critical in determining whether trait affect should be the focus of psychosocial treatments such as cognitive–behavioural therapy for symptoms of the schizophrenia. Results also highlight the nuance that differentiating discrete emotions can add. Further, the present observations suggest that, although trait emotion abnormalities are implicated in both the prodrome and then later after onset, this critical domain may need to be assessed and conceptualised differently across stages of psychosis. Although examining trait emotion abnormalities in the prodrome is still in its early stages, early evidence highlights a hefty role for secondary influences. Experimental studies and large-scale longitudinal investigations will be instrumental in improving our understanding of this important area, and for driving early identification and treatment development efforts.

## Supplementary material

For supplementary material accompanying this paper visit http://dx.doi.org/10.1192/bjo.2019.64. click here to view supplementary material
