# Supplementary material for: Trait emotional experience in individuals with schizophrenia and youth at clinical high risk for psychosis
Source: BJPsych Open. 2019 Sep 10;5(5):e78. doi: 10.1192/bjo.2019.64 (PMC6737516; doi:10.1192/bjo.2019.64)
Supplement: Supplementary file 1 [file S2056472419000644sup001.docx]

**Table 1.** Correlations between affect and negative symptoms for Sample 2.

| Symptom Type |  | Positive  Affect | Negative Affect |
| --- | --- | --- | --- |
| SIPS Negative Total |  | -.44^**^ | .60^***^ |
|  |  |  |  |
| 2 Dimensions |  |  |  |
|  | Diminished Emotion | -.48^***^ | .51^***^ |
|  | Diminished Volition | -.40^**^ | .61^***^ |
|  |  |  |  |
| SIPS Negative |  |  |  |
|  | Anhedonia | -.25+ | .41^**^ |
|  | Avolition | -.45^**^ | .46^**^ |
|  | Emotion Expression | -.31^*^ | .48^***^ |
|  | Emotion Experience | -.45^**^ | .65^***^ |
|  | Ideational Richness | -0.13 | .34^*^ |
|  | Occupational Functioning | -.45^**^ | .50^***^ |

**Table 2.** Correlations between affect and negative symptoms for Sample 1.

| Symptom Type |  | Positive  Affect | Negative Affect |
| --- | --- | --- | --- |
| SANS Total |  | -.33^*^ | 0.18 |
|  |  |  |  |
| 2 Dimensions |  |  |  |
|  | Diminished Expressivity | -.25^+^ | 0.08 |
|  | Diminished Motivation | -.36^**^ | .28^*^ |
|  |  |  |  |
| NIMH Domains |  |  |  |
|  | Blunted Affect | -.25^+^ | -0.08 |
|  | Alogia | -0.19 | .24^+^ |
|  | Avolition | -.39^**^ | 0.23 |
|  | Anhedonia | -.29^*^ | .28^*^ |
|  | Asociality | -.28^*^ | .24^+^ |

**Table 3.** Correlations between affect and negative symptoms for Sample 2.

[AUTHOR TO SPLLY MISSING TABLE]

**Table 4**. Correlations between affect and negative symptoms while controlling for depression, anxiety, and antipsychotics in Sample 1.

| Symptom Type |  | Positive  Affect | Negative Affect |
| --- | --- | --- | --- |
| SANS Total |  | -.32^*^ | 0.2 |
|  |  |  |  |
| 2 Dimensions |  |  |  |
|  | Diminished Expression | -0.21 | 0.13 |
|  | Diminished Motivation | -.40^**^ | 0.22 |
|  |  |  |  |
| NIMH Domains |  |  |  |
|  | Blunted Affect | -0.19 | 0.01 |
|  | Alogia | -0.19 | 0.24 |
|  | Avolition | -.45^**^ | 0.22 |
|  | Anhedonia | -.34^*^ | 0.2 |
|  | Asociality | -.29^*^ | 0.2 |

**Table 5.** Correlations between affect and negative symptoms while controlling for depression and anxiety in Sample 2.

| Symptom Type |  | Positive  Affect | Negative Affect |
| --- | --- | --- | --- |
| SIPS Negative Total |  | -0.16 | .42^**^ |
|  |  |  |  |
| 2 Dimensions |  |  |  |
|  | Diminished Emotion | -0.2 | 0.22 |
|  | Diminished Volition | -0.13 | .46^**^ |
|  |  |  |  |
| SIPS Negative |  |  |  |
|  | Anhedonia | -0.01 | 0.21 |
|  | Avolition | -0.16 | 0.13 |
|  | Emotion Expression | -0.21 | .50^***^ |
|  | Emotion Experience | -0.12 | .43^**^ |
|  | Ideational Richness | -0.03 | .34^*^ |
|  | Occupational Functioning | -0.2 | .28^+^ |
